# The association between blood cadmium level, frequency and amount of *gejang* (marinated crab) intake

**DOI:** 10.1186/s40557-016-0109-0

**Published:** 2016-05-14

**Authors:** Chang Yul Choi, Gun Il Park, Young Seok Byun, Man Joong Jeon, Kwang Hae Choi, Joon Sakong

**Affiliations:** Department of Occupational and Environmental Medicine, Yeungnam University Hospital, 170, Hyeonchung-ro, Nam-gu, Daegu, 705-802 Republic of Korea; Department of Preventive Medicine and Public Health, College of Medicine, Yeungnam University, 170, Hyeonchung-ro, Nam-gu, Daegu, 705-802 Republic of Korea; Department of Pediatrics Medicine, Yeungnam University Hospital, 170, Hyeonchung-ro, Nam-gu, Daegu, 705-802 Republic of Korea

**Keywords:** *Gejang* (marinated crab), Cadmium, KNHANES

## Abstract

**Background:**

*Gejang* (marinated crab) is a favorite traditional food and a main source of crab intake among Koreans. The present study aimed to identify the possibility of cadmium inflow to the body through *gejang*; accordingly, the relationship between *gejang* intake frequency and amount, and blood cadmium concentrations was investigated.

**Methods:**

Using data from the first Korea National Health and Nutrition Examination Survey in the sixth period in 2013, means and standard errors of blood cadmium concentrations in relation to *gejang* intake frequency and amount, as well as the monthly intake of *gejang*, were obtained from 1381 participants for whom data regarding blood cadmium concentration measurements was available.

**Results:**

After adjustment for confounding factors, a *gejang* intake frequency of four or fewer times per week and a monthly intake of 200 cm^3^ or less had no significant effect on blood cadmium concentrations. However, participants with *gejang* intake of at least five times per week had a weighted mean cadmium concentration of 2.12 μg/L (*p* < 0.001), and participants who had a *gejang* monthly intake of more than 200 cm^3^ had a weighted mean cadmium concentration of 1.76 μg/L (*p* < 0.001).

**Conclusion:**

These results suggest that to minimize the effect of *gejang* consumption on blood cadmium level, *gejang* intake should be limited to four or less times per week and 200 cm^3^ or less per month. Weekly intake of at least five times and monthly intake of more than 200 cm^3^ may increase blood cadmium levels.

## Background

Cadmium, a representative hazardous metal to the body, enters the body through environmental exposures, including occupational exposures in industrial fields, air, water, and soil; it also enters through personal exposures, including foods or smoking [1]. Absorption of large amounts of cadmium from foods causes nausea, vomiting, stomachache, stomach cramps, and diarrhea [2]. Moreover, long-term intake of cadmium leads to reabsorption disorder in the renal proximal tubules, excretion of low molecular weight proteins, aminoaciduria, dextrosuria, phosphaturia, and even glomerular damage in severe cases [3], which sometimes results in osteomalacia, osteoporosis, fracture, and reduced bone density.

Environmental cadmium is generated by natural activities such as volcanic activity or erosion, mine developments, metal productions, fossil fuel combustions, sewage residues, garbage incinerations, and phosphatic fertilizers [4], and it flows to the sea, resulting in cadmium exposure of marine organisms [5]. Of marine organisms, epithelial cells of crustaceans are composed of lipid bilayer structures with hydrophobic interiors and hydrophilic exteriors, so that pollutants including cadmium are mainly accumulated in internal organs such as gill, liver, and pancreas of crabs through transport proteins or ion pumps [6].

In 2013, the Food Standards Agency of the UK (2013) reported that cadmium concentrations in crabs were as high as 26 mg/kg in internal organs of crabs and 0.14 mg/kg in muscle meat of crab legs and claws, showing that, among marine organisms, crustaceans can accumulate cadmium [7].

According to an investigation of heavy metal concentrations in fishery products in South Korea, cadmium concentrations in blue crabs were as high as 10.43 mg/kg in internal organs of crabs and 0.439 mg/kg in meat of crab legs and claws, and those in snow crabs were as high as 45.46 mg/kg in internal organs of crabs and 0.20 mg/kg in meat of crab legs and claws (Institute For Environment & Community, 2010) [8]. Since these values represent more than dozens of times the permissible concentration of cadmium in mollusks (2 mg/kg), it has caused social ramifications.

Korean people directly consume internal organs of crabs as *yangnyeom gejang* (spicy marinated crab), *ganjang gejang* (soy sauce marinated crab), and as mixed internal organs of crabs with rice. Thus, Koreans may have a higher chance of cadmium inflow into the body through *gejang* than do people in other countries. Therefore, to establish safe intake limits and to decrease anxiety regarding *gejang* intake, it is necessary to investigate whether *gejang* intake provides a significant inflow path for cadmium.

Therefore, this study investigated the relationships between *gejang* intake frequency and amount, and cadmium concentrations using Korea National Health and Nutrition Examination Survey data.

## Subjects and methods

### Research subjects

The Korea National Health and Nutrition Examination Survey is one of the representative surveys in South Korea; it is conducted to investigate health levels in association with intakes of foods and nutrition by Korean people and is led by the Ministry of Health and Welfare. After the fourth survey from 2007–2009, the survey has been conducted annually. For the sampling design, using the first year data (conducted in 2013) of the sixth period, a two-step stratified cluster sampling method was used with data from the population and housing census in 2010 as a sampling frame. A total of 192 communities, the first sampling unit, were sampled based on the first stratification standard (city and providence; dong, eup, and myeon [Korean administrative units]; house type), the second stratification standard (ratio of residential area), and internal stratification standard (education level of heads of household). In addition, a total of 20 households, the second sampling unit, were sampled within the sample community using a systematic sampling method. A total of 3840 sample households within 192 sampled communities nationwide were selected as research subjects. The samples were weighted to closely reflect the actual health behaviors and levels of the Korean people.

The Korea National Health and Nutrition Examination Survey is composed of a health survey, examination survey, and nutrition survey. The health and examination surveys were conducted in a mobile examination center, and the nutrition survey was performed by directly visiting subject households. Of a total of 10,113 survey subjects in the first year in the 6^th^ period (2013), 8018 participated in at least one of the three surveys, which corresponded to a 79.3 % participation rate [9]. Of these, 2355 people had results for blood cadmium measurements, of which 1381 people were finally selected as subjects for analysis, excluding 974 people (913 for not having the nutrition survey and 61 for having a positive smoking status) who missed each survey variable.

## Methods

Intake frequencies of rice and *gejang* were investigated using the results of a food frequency questionnaire survey. Intake frequencies were divided into nine categories including “eat rarely,” “eat once monthly,” “eat 2–3 times monthly,” “eat once weekly,” “eat 2–4 times weekly,” “eat 5–6 times weekly,” “eat once daily,” “eat twice daily,” and “eat thrice daily”. The mean single-serving intake amount of *gejang* was divided into “eat rarely,” “eat 1 teaspoon,” “1 table spoon,” and “1/4 cup.” Independent variables for intake frequency of *gejang* were again divided into six groups, including “eat rarely,” “eat once monthly,” “eat 2–3 times monthly,” “eat once weekly,” “eat 2–4 times weekly,” and “eat 5–6 times weekly”. Moreover, considering that 1 teaspoon corresponds to 5 cm^3^ and 1 table spoon to 15 cm^3^, independent variables for a single-serving time intake amount of *gejang* were re-divided into four groups, including “eat rarely, “5 cm^3^,” “15 cm^3^,” and “50 cm^3^.” In addition, monthly *gejang* intake amount was calculated by multiplying the intake frequency by the single-serving intake amount; the monthly intake amount was divided into “eat rarely,” “eat 100 cm^3^ or less per month,” “eat 101–200 cm^3^ per month,” and “eat more than 200 cm^3^ per month”.

Age groups were divided into five groups: 20–29 years, 30–39 years, 40–49 years, 50–59 years, and 60 years or older. Education level was divided into three groups: “middle school graduation or lower,” “high school graduation,” and “college or higher (including community colleges)”. Mean monthly household income was calculated using equalized income (=total income/sqrt of number of household members) and was classified by quartiles into “low,” “mid-low,” “mid-high” and “high”. Smoking status was divided into three groups: “never smoker,” “former smoker,” and “current smoker”. Level of alcohol consumption was divided into four groups: “never,” “one time or less per month,” “2–4 times weekly,” and “at least once weekly”.

### Statistical analysis

Since the survey investigated samples through stratification, statistical analysis was performed considering stratification variables and weighting in order to identify the effect of complex sample design, in which variables that can function as confounding factors such as sex, age, smoking status, and rice intake were adjusted before the analysis. Socio demographic characteristics of subjects were analyzed using the chi-square test, and one-way analysis of variance (ANOVA) was used to compare blood cadmium concentrations depending on *gejang* intake.

For analysis of the weighted complex sample design, the complex sample command included in the SPSS 22.0 statistical package was used to estimate frequency of the parent population from the sample survey data. By setting variables corresponding to the first sampling unit, stratification variables, and weighting, estimated frequency values of parent population and standard errors were calculated using a statistical algorithm. Blood cadmium concentrations depending on *gejang* intake of the parent population were compared using a one-way ANOVA for a complex sample. In addition, sex, age, smoking status, and rice intake were set as control variables, and then blood cadmium concentrations of the parent population depending on *gejang* intake were compared using complex sample analysis of covariance (ANCOVA).

## Results

General characteristics of subjects by sex are presented in Table 1. The mean age of the 1381 subjects was 42 years, and 627 (45.4 %) were males. Males and females in their 20s accounted for 24.7 and 21.9 %, respectively, whereas those in their 60s accounted for 10.5 and 9.8 %, respectively. The highest proportion of education level was high school graduation in both males and females, at 43.1 and 41.0 %, respectively. The proportions of smokers and never-smokers were 23.2 and 60.6 %, respectively. Alcohol consumption of more than 1 time per week had the highest frequency in males, accounting for 35.2 %, whereas consumption of alcohol one or fewer times per month had the highest frequency for females, accounting for 43.0 %.

**Table 1:** General characteristics of subjects

				N(%)
Characteristics	Total	Male	Female	*p*-value*
Sex	1381 (100.0)	627 (45.4)	754 (54.6)	
Age (yr)				
20–29	320 (23.2)	155 (24.7)	165 (21.9)	0.709
30–39	313 (22.7)	136 (21.7)	177 (23.5)	
40–49	303 (21.9)	133 (21.2)	170 (22.5)	
50–59	305 (22.1)	137 (21.9)	168 (22.3)	
≥60	140 (10.1)	66 (10.5)	74 (9.8)	
Education				
Middle school or less	252 (18.3)	94 (15.0)	158 (21.0)	0.015
High school	579 (41.9)	270 (43.1)	309 (41.0)	
College or more	549 (39.8)	263 (41.9)	286 (38.0)	
Income^a^				
Low	344 (25.0)	156 (24.9)	188 (25.0)	0.854
Mid-low	344 (25.0)	163 (26.0)	181 (24.1)	
Mid-high	345 (25.0)	154 (24.6)	191 (25.4)	
High	345 (25.0)	153 (24.5)	192 (25.5)	
Smoking				
Never	837 (60.6)	164 (26.2)	673 (89.3)	<0.001
Former	224 (16.2)	192 (30.6)	32 (4.2)	
Current	320 (23.2)	271 (43.2)	49 (6.5)	
Alcohol consumption (n)				
Never	186 (14.5)	60 (9.8)	126 (18.8)	<0.001
≤1/month	418 (32.6)	130 (21.2)	288 (43.0)	
2-4/month	383 (29.8)	207 (33.8)	176 (26.3)	
≥1/week	296 (23.1)	216 (35.2)	80 (11.9)	

*Calculated by chi-square test

^a^Mean monthly household income was classified into quartiles

Blood cadmium concentrations of subjects depended on *gejang* intake frequency, single-serving *gejang* intake amount, and monthly *gejang* intake amount (Table 2). There were no significant differences in blood cadmium concentrations of subjects based on *gejang* intake frequency and single-serving intake amount. There was also no significant difference in blood cadmium concentration based on monthly *gejang* intake. Furthermore, when research subjects were divided by sex, neither males nor females showed a significant difference in blood cadmium concentrations depending on *gejang* intake frequency, single-serving intake amount, and monthly intake amount.

**Table 2:** Blood cadmium concentrations by *gejang* (marinated crab) intake frequency and amount (unweighted and weighted)

	Unweighted						Weighted					
Characteristics	Male		Female		Total		Male		Female		Total	
	Number^a^	Cd^b^	Number^a^	Cd^b^	Number^a^	Cd^b^	Number^c^	Cd^b^	Number^c^	Cd^b^	Number^c^	Cd^b^
Frequency												
Rarely	514	0.85 ± 0.03	670	1.04 ± 0.02	1184	0.96 ± 0.02	12,548,740	0.84 ± 0.03	14,304,696	1.07 ± 0.03	26,853,437	0.96 ± 0.02
1/month	72	1.02 ± 0.15	59	1.08 ± 0.08	131	1.05 ± 0.09	1,788,377	1.00 ± 0.12	1,345,577	1.14 ± 0.09	3,133,955	1.06 ± 0.08
2–3/month	29	0.94 ± 0.08	17	1.12 ± 0.13	46	1.00 ± 0.07	735,207	0.95 ± 0.09	269,466	1.07 ± 0.13	1,004,674	0.98 ± 0.08
1/week	11	1.06 ± 0.18	4	1.40 ± 0.17	15	1.15 ± 0.14	218,089	1.03 ± 0.16	65,181	1.44 ± 0.15	283,271	1.13 ± 0.14
2–4/week	-	-	3	1.09 ± 0.11	3	1.09 ± 0.11	-	-	43,525	1.15 ± 0.01	43,525	1.15 ± 0.01
5–6/week	1	1.82	1	2.26	2	2.04 ± 0.22	13,692	1.82	30,364	2.26	44,056	2.12 ± 0.13
*p*-value*		0.217		0.340		0.149		<0.001		<0.001		<0.001
Intake per meal												
Rarely	514	0.85 ± 0.03	670	1.04 ± 0.02	1184	0.96 ± 0.02	12,548,740	0.84 ± 0.03	14,304,696	1.07 ± 0.03	26,853,437	0.96 ± 0.02
5 cm^3^	5	0.61 ± 0.10	6	1.22 ± 0.24	11	0.94 ± 0.16	91,884	0.63 ± 0.10	124,635	1.30 ± 0.18	216,520	1.02 ± 0.17
15 cm^3^	36	1.11 ± 0.30	25	1.16 ± 0.12	61	1.13 ± 0.18	914,111	1.04 ± 0.22	514,450	1.30 ± 0.16	1,428,562	1.13 ± 0.15
50 cm^3^	72	0.99 ± 0.06	53	1.09 ± 0.07	125	1.03 ± 0.05	1,749,370	0.99 ± 0.07	1,115,030	1.08 ± 0.08	2,864,400	1.02 ± 0.05
*p*-value*		0.099		0.689		0.212		<0.001		<0.001		<0.001
Intake per month												
Rarely	514	0.85 ± 0.03	670	1.04 ± 0.02	1184	0.96 ± 0.02	12,548,740	0.84 ± 0.03	14,304,696	1.07 ± 0.03	26,853,437	0.96 ± 0.02
≤ 100 cm^3^	82	0.99 ± 0.14	66	1.11 ± 0.07	148	1.04 ± 0.08	2,032,772	0.96 ± 0.11	1,446,455	1.15 ± 0.08	3,479,227	1.04 ± 0.07
101–200 cm^3^	30	1.04 ± 0.09	16	1.09 ± 0.09	46	1.05 ± 0.07	708,902	1.05 ± 0.08	250,769	1.06 ± 0.11	959,672	1.06 ± 0.06
> 200 cm^3^	1	1.82	2	1.71 ± 0.55	3	1.75 ± 0.32	13,692	1.82	56,890	1.75 ± 0.39	70,583	1.76 ± 0.31
*p*-value*		0.137		0.384		0.088		<0.001		<0.001		<0.001

^a^Number of consumers of 6^th^ Korea National Health and Nutritional Examination Survey (KNHANES) per individual category

^b^Blood cadmium concentrations were expressed as mean ± standard error, unit: μg/L

^c^Estimated number of the Korean population per individual category

*Calculated by ANCOVA

When blood cadmium concentrations of subjects were estimated by weighting and blood cadmium concentration of all subjects, *gejang* intake frequency tended to statistically significantly increase; however, the increase in cadmium concentration was clinically significant at an intake frequency of 2–4 times or fewer per week. However, the group with a 5–6 times weekly intake tended to show a clinically significant increase in cadmium concentration.

In addition, blood cadmium concentrations of all subjects tended to statistically significantly increase with monthly *gejang* intake; however, the group with a monthly intake of 200 cm^3^ or less did not demonstrate a clinically significant increase in cadmium concentration, although there was a trend for such an increase.

Table 3 presents estimated blood cadmium concentrations depending on *gejang* intake frequency, single-serving intake amount, and monthly intake amount after weighting and adjusting confounding factors. Blood cadmium concentrations of all subjects showed a trend of a statistically significant increase with increased monthly *gejang* intake; the group with a monthly intake of 200 cm^3^ or less did not have a clinically significant increase in cadmium concentration. In contrast, the group with a monthly intake of more than 200 cm^3^ intake tended to have an increase in cadmium concentration.

**Table 3:** Weighted and adjusted blood cadmium concentrations by *gejang* (marinated crab) intake frequency and amount

Characteristics	Cadmium concentration (μg/L)^a^
Frequency	
Rarely	0.97 ± 0.02
1/month	1.04 ± 0.07
2–3/month	0.99 ± 0.06
1/week	1.10 ± 0.09
2–4/week	0.98 ± 0.16
5–6/week	1.65 ± 0.13
*p*-value*	<0.001
Intake per meal	
Rarely	0.97 ± 0.02
5 cm^3^	0.93 ± 0.10
15 cm^3^	1.13 ± 0.14
50 cm^3^	1.00 ± 0.04
*p*-value*	0.467
Intake per month	
Rarely	0.97 ± 0.02
≤ 100 cm^3^	1.03 ± 0.06
101–200 cm^3^	1.05 ± 0.06
> 200 cm^3^	1.46 ± 0.18
*p*-value*	0.016

^a^Weighted and adjusted for sex, age, smoking and rice consumption

*Calculated by ANCOVA

## Discussion

This study found that a *gejang* weekly intake of four times or fewer and a monthly intake of 200 cm^3^ or less had no significant effects on changes in blood cadmium concentrations. However, a weekly *gejang* intake of five times or more resulted in a weighted mean cadmium concentration as high as 2.12 μg/L (*p* < 0.001). Moreover, a higher weighted mean cadmium concentration of 1.76 μg/L (*p* < 0.001) was observed in those with a monthly *gejang* intake of more than 200 cm^3^.

In the UK, it has been reported that mean cadmium concentrations of internal organs and muscle of legs and claws were 4.00 mg/kg and 0.27 mg/kg, respectively [7]. When 33 Mediterranean crab samples were investigated in Italy, mean cadmium concentrations in internal organs and muscles of legs and claws were 1.19 mg/kg and 0.01 mg/kg, respectively [4]. It was reported that the cadmium concentration in internal organs of Atlantic spider crab measured in the US in 2010 was 2 mg/kg, whereas muscle of legs and claws of crabs had an extremely small amount of cadmium [10]. In an evaluation of heavy metal concentrations in internal organs and muscles of legs and claws of crabs by Mok et al. conducted in South Korea in 2010, the cadmium value measured in internal organs of crabs was dozens of times higher than that of muscles of legs and claws. In addition, the study reported that internal organs contained 95 % of the total cadmium in crabs [11]. As such, various studies have demonstrated that cadmium concentrations measured in internal organs of crabs were higher than those of muscles of legs and claws. Since Korean people directly eat internal organs of crabs as *yangnyeom gejang* and *ganjang gejang*, they have a higher risk of exposure to cadmium than western people who normally eat muscles of legs and claws of crabs.

Many countries began to be interested in investigating food pollution status and establishing preventive measures [12, 13], and a joint meeting with the United Nations Food and Agriculture Organization and the World Health Organization decided to perform a pollutant monitoring project in foods. In South Korea, the Korea Food and Drug Administration has been performing a heavy metal monitoring project on fishery products, agricultural products, livestock products, and processed foods continuously since 2000.

The Provisional Tolerable Monthly Intake (PTMI) recommended by the Joint FAO/WHO Expert Committee on Food Additives is a representative safety guideline for dietary exposure to pollutants. As of 2010, the provisional monthly intake limit of cadmium was 25 μg/kg body weight/month [14].

The Korea Food and Drug Administration conducted a study to evaluate exposure/hazard of foods. When food intake amounts and body weights were analyzed using the data of the second National Health and Nutrition Survey (2008) in the fourth period published by the Korea Centers for Disease Control and Prevention [15], the mean cadmium intake amount through all foods was 10.4 μg/day, corresponding to 22.7 % of PTMI, of which the intake amount through crustaceans was 0.1 μg/day, corresponding to 0.3 % of PTMI [16], indicating that it was negligible.

However, since the mean cadmium intake amount included data from a number of people who rarely eat crustaceans and *gejang*, it was possible to have underestimated dietary exposure levels of frequent eaters of *gejang*. Thus, cadmium concentrations of people should be compared and evaluated according to intake frequency and intake amount of *gejang*.

The present study needs to be interpreted considering the following limitations. First, blood heavy metal concentrations are affected not only by recent food intakes, but also by long-term accumulations, but analyses for each factor were performed only considering current exposure. Second, this study did not exclude people who had either direct occupational exposures or other overexposure-prone procedures. However, the effect is considered negligible because the number of corresponding people was expected to be small. Third, since this study did not reflect accurate *gejang* intake amounts, it should be determined more accurately; furthermore, to better analyze the relationships between *gejang* intake and blood cadmium concentrations, hair analysis needs to be performed to identify the long-term heavy metal exposure, in addition to blood cadmium concentrations. Fourth, the number of people who had a high *gejang* intake was small. Since this study is a cross-sectional study based on the pre-existing KNHANES VI-1 data, where only three subjects were found to intake *gejang* >200 cm^3^, it is difficult to draw conclusions by proposing a guidance value of *gejang* intake. Therefore, well-designed further studies are strongly suggested.

## Conclusions

The results of this study demonstrate that weekly intake of at least five times and monthly intake of more than 200 cm^3^ may increase blood cadmium levels. Therefore, to minimize the effect of *gejang* consumption on blood cadmium level, it is recommendable that *gejang* intake should be limited to four times or less per week and 200 cm^3^ or less per month.
